# Supplementary material for: Screening of targeted panel genes in Brazilian patients with primary ovarian insufficiency
Source: PLoS One. 2020 Oct 23;15(10):e0240795. doi: 10.1371/journal.pone.0240795 (PMC7584253; doi:10.1371/journal.pone.0240795)
Supplement: S1 Table — (DOCX) [file pone.0240795.s001.docx]

**Supplemental Table 1 – Analysis of identified variants *in silico* prediction tools**

| **Chr** | **Start** | **cytoBand** | **Gene** | **dbSNP** | **SIFT** | **Polyphen2** | **MutationTaster** | **CADD_phred** | **GERP** |
| --- | --- | --- | --- | --- | --- | --- | --- | --- | --- |
| 1 | 40228799 | 1p34.2 | *BMP8B* | rs149276444 | D | P | D | 24.8 | 3.75 |
| 15 | 83240214 | 15q25.2 | *CPEB1* | rs200188266 | D | D | D | 24.2 | 4.7 |
| 19 | 17932264 | 19p13.11 | *INSL3* | rs200056709 | T | P | N | 22.2 | -0.632 |
| 6 | 119136196 | 6q22.31 | *MCM9* | rs61744508 | T | B | N | 9.988 | 3.28 |
| 6 | 119137360 | 6q22.31 | *MCM9* | rs1046135510 | T | B | N | 7.391 | -0.743 |
| 8 | 11566101 | 8p23.1 | *GATA4* | rs780764610 | T | B | D | 7.844 | 1.43 |
| 5 | 132197863 | 5q31.1 | *GDF9* | rs1216260561 | NA | NA | NA | NA | NA |
| 6 | 42652599 | 6p21.1 | *UBR2* | rs1017000245 | D | D | D | 24.2 | 5.64 |
| 11 | 108106399 | 11q22.3 | *ATM* | rs146382972 | D | P | N | 23 | 4.71 |
| 11 | 108200946 | 11q22.3 | *ATM* | rs147604227 | T | P | D | 23.9 | 3.79 |
| 7 | 99780415 | 7q22.1 | *STAG3* | Absent | NA | NA | NA | NA | NA |
| 7 | 99798481 | 7q22.1 | *STAG3* | Absent | NA | NA | D | 35 | -0.991 |
| X | 50658771 | Xp11.22 | *BMP15* | rs782799707 | NA | NA | D | 32 | 1.53 |
| 6 | 119177696 | 6q22.31 | *MCM9* | rs545524695 | D | P | D | 28.7 | 6.11 |
| 2 | 203407114 | 2q33.2 | *BMPR2* | Absent | D | D | D | 33 | 5.41 |
| 3 | 16636081 | 3p24.3 | *DAZL* | Absent | NA | NA | A | 44 | 6.02 |
| 1 | 40230385 | 1p34.2 | *BMP8B* | rs199806017 | D | D | N | 24.6 | 2.1 |
| 6 | 106553285 | 6q21 | *PRDM1* | rs200035233 | D | D | D | 14.33 | 3.5 |
| 2 | 49190662 | 2p16.3 | *FSHR* | rs763676828 | D | D | D | 27.2 | 5.64 |
| 2 | 49216133 | 2p16.3 | *FSHR* | rs746673169 | NA | NA | NA | NA | NA |
| 22 | 31840659 | 22q12.2 | *EIF4ENIF1* | rs374538489 | T | D | D | 13.79 | 4.73 |
| 7 | 144098504 | 7q35 | *NOBOX* | rs372037920 | T | P | N | 0.022 | 0.745 |
| 8 | 11615875 | 8p23.1 | *GATA4* | rs115099192 | D | D | D | 25.8 | 4.78 |
| 5 | 132199837 | 5q31.1 | *GDF9* | Absent | T | B | N | 10.69 | 4.43 |
|  |  |  |  |  |  |  |  |  |  |
| **Chr** | **Start** | **cytoBand** | **Gene** | **dbSNP** | **SIFT** | **Polyphen2** | **MutationTaster** | **CADD_phred** | **GERP** |
| 5 | 176311048 | 5q35.2 | *HK3* | rs376092049 | D | D | D | 26.3 | 4.76 |
| 6 | 31138265 | 6p21.33 | *POU5F1* | Absent | T | B | D | 22.9 | 0.826 |
| X | 101092587 | Xq22.1 | *NXF5* | rs113591248 | T | B | N | 10.19 | 0.794 |
| X | 101096484 | Xq22.1 | *NXF5* | rs142009656 | T | B | N | 0.025 | 2.18 |
| X | 101096741 | Xq22.1 | *NXF5* | rs113468014 | T | B | N | 0.001 | -3.71 |
| 8 | 11566101 | 8p23.1 | *GATA4* | rs780764610 | T | B | D | 7.844 | 1.43 |
| 8 | 90992986 | 8q21.3 | *NBN* | rs201816949 | D | D | D | 28.7 | 5.75 |
| 11 | 108181003 | 11q22.3 | *ATM* | rs587782503 | D | D | D | 34 | 5.7 |
| X | 101092588 | Xq22.1 | *NXF5* | rs140252282 | NA | NA | A | 35 | 2.42 |
| X | 107422058 | Xq22.3 | *COL4A6* | rs143895379 | D | D | D | 24.2 | 4.67 |
| X | 128884450 | Xq26.1 | *XPNPEP2* | rs138365897 | D | D | D | 23.4 | 4.26 |
| 1 | 115401309 | 1p13.2 | *SYCP1* | Absent | D | D | D | 32 | 5.11 |
| X | 101095822 | Xq22.1 | *NXF5* | Absent | T | P | D | 23.6 | 2.05 |
| 5 | 176310798 | 5q35.2 | *HK3* | rs199684264 | D | P | D | 19.7 | 3.5 |
| 6 | 31138311 | 6p21.33 | *POU5F1* | rs2000769740 | D | D | D | 33 | 3.7 |
| 2 | 58386928 | 2p16.1 | *FANCL* | rs759217526 | NA | NA | NA | NA | NA |
| 11 | 108121465 | 11q22.3 | *ATM* | rs769214234 | T | B | N | 1.33 | -2.61 |
| 19 | 45858047 | 19q13.32 | *ERCC2* | rs142568756 | D | D | D | 34 | 5.35 |
| 7 | 144098983 | 7q35 | *NOBOX* | rs77587352 | T | D | N | 13.3 | -1.98 |
| 5 | 176317836 | 5q35.2 | *HK3* | rs141123858 | D | D | D | 25.8 | 4.79 |
| 5 | 140076899 | 5q31.3 | *HARS2* | rs61736946 | D | D | D | 24 | 5.67 |
| 22 | 45757744 | 22q13.31 | *SMC1B* | rs199797179 | D | D | D | 29.8 | 1.85 |
| 1 | 115466098 | 1p13.2 | *SYCP1* | rs147626229 | D | B | N | 23.2 | 2.56 |
| 5 | 132200035 | 5q31.1 | *GDF9* | rs751002918 | D | B | N | 23.9 | 5.14 |
| 2 | 58386928 | 2p16.1 | *FANCL* | rs759217526 | NA | NA | NA | NA | NA |
| 4 | 84337987 | 4q21.23 | *HELQ* | rs761786816 | NA | NA | NA | NA | NA |

Complete *in silico* scores and predictions are available on ANNOVAR (<https://annovar.openbioinformatics.org/>). D: deleterious or disease-causing or probably damaging; P: possible damaging; N: neutral; T: tolerated; B: benign; GERP: >2, deleterious; CADD: >13, deleterious; NA: non-available.
